# A New Approach to Design Autonomous Wireless Sensor Node Based on RF Energy Harvesting System

**DOI:** 10.3390/s18010133

**Published:** 2018-01-05

**Authors:** Alex Mouapi, Nadir Hakem

**Affiliations:** Underground Communication Research Laboratory, University of Québec in Abitibi-Témiscamingue, 675, 1e avenue, Val-d’Or, QC J9P1Y3, Canada; nadir.hakem@uqat.ca

**Keywords:** energy model, gradient method search, ISM band, LEACH protocol, Radio-frequency Energy Harvesting System, Wireless Sensor Network

## Abstract

Energy Harvesting techniques are increasingly seen as the solution for freeing the wireless sensor nodes from their battery dependency. However, it remains evident that network performance features, such as network size, packet length, and duty cycle, are influenced by the sum of recovered energy. This paper proposes a new approach to defining the specifications of a stand-alone wireless node based on a Radio-frequency Energy Harvesting System (REHS). To achieve adequate performance regarding the range of the Wireless Sensor Network (WSN), techniques for minimizing the energy consumed by the sensor node are combined with methods for optimizing the performance of the REHS. For more rigor in the design of the autonomous node, a comprehensive energy model of the node in a wireless network is established. For an equitable distribution of network charges between the different nodes that compose it, the Low-Energy Adaptive Clustering Hierarchy (LEACH) protocol is used for this purpose. The model considers five energy-consumption sources, most of which are ignored in recently used models. By using the hardware parameters of commercial off-the-shelf components (Mica2 Motes and CC2520 of Texas Instruments), the energy requirement of a sensor node is quantified. A miniature REHS based on a judicious choice of rectifying diodes is then designed and developed to achieve optimal performance in the Industrial Scientific and Medical (ISM) band centralized at 2.45 GHz. Due to the mismatch between the REHS and the antenna, a band pass filter is designed to reduce reflection losses. A gradient method search is used to optimize the output characteristics of the adapted REHS. At 1 mW of input RF power, the REHS provides an output DC power of 0.57 mW and a comparison with the energy requirement of the node allows the Base Station (BS) to be located at 310 m from the wireless nodes when the Wireless Sensor Network (WSN) has 100 nodes evenly spread over an area of $300\;\times \;300\;m^{2}$ and when each round lasts 10 min. The result shows that the range of the autonomous WSN increases when the controlled physical phenomenon varies very slowly. Having taken into account all the dissipation sources coexisting in a sensor node and using actual measurements of an REHS, this work provides the guidelines for the design of autonomous nodes based on REHS.

## 1. Introduction

Nowadays, the concept of the smart city is increasingly used to refer to the integration of Information and Communications Technology (ICT) in the urban environment. Thus, the Wireless Sensors Network (WSN), public access terminals in wireless (WI-FI), smart metering and applications for smartphones are innovations that facilitate people’s daily lives. In particular, WSNs are increasingly used in various areas, including but not limited to vehicle tracking [1], structural monitoring [2], habitat monitoring [3], and health monitoring [4]. This revolutionary trend can be primarily attributed to the recent advances in the integration of Micro Electro Mechanical System (MEMS) [5], low-power circuit design [6], and wireless technology [7] into a small form factor for low-cost mass production. Paradoxically, the explosive growth in low-consumption mobile devices, and batteries, which initially contributed to their launch, has since become a brake on their development, particularly because of maintenance problems (refill or replacement) associated with them. This is even more problematic when the sensor nodes are deployed in hard-to-reach places or intended to operate for much longer duration (for instance, years) following their deployment. This is, for example, the case of applications such as monitoring of buildings and structural health [2], in which the batteries are difficult or impossible to replace. In an effort to increase the energy autonomy of sensor nodes, energy harvesting techniques have recently been proposed as a battery replenishment solution [8].

Typically, energy harvesting calls for recovery of a primary energy source, in the immediate environment of the sensor node, to turns it into electrical energy capable of powering the node. These techniques differ from one another due to the nature of the primary energy source. The main sources of energy considered in the literature are mechanical vibrations, internal light, the sun, wind, electromagnetic waves, heat, etc. [8]. Unlike primary sources that show an intermittent character depending on the seasons (wind, sun, heat), operation of the mechanical machinery (vibrations), time (internal light), and electromagnetic waves show a more constant presence in light of the extension of telecommunications systems. Also, applications such as the Internet of the Things (IoT) allow for the incorporation of several sensor nodes into the same building, which enables presence detection, and monitoring of light, temperature, and other environmental conditions [9]. For such applications, it would be difficult to have a source of primary energy other than radio-frequency waves capable of safely supplying the nodes incorporated into the building. Furthermore, in previous work [10], a state-of-the art survey of energy harvesting techniques showed that RF energy harvesters in these more constant energy sources have smaller dimensions compared to other harvesters; this is another advantage for miniature applications.

The main objective of this work is to achieve long life for sensor nodes for applications in remote and especially difficult-to-access environments. The proposed solution is to enslave the node to the ubiquitous RF energy source. Enslavement involves determining the type of physical phenomenon that can be measured, network size, and the maximum size of the transmitted data. A state-of-the art survey of previous works is provided in Section 2. Note that the slave sensor node consists of two parts: the sensor node and the REHS. Thus, in Section 3, a comprehensive energy model of consumption of a sensor node is proposed. A methodology for designing a miniature REHS optimized to achieve better performance is featured in Section 4. The electrical output characteristics of the RF micro generator are then used to evaluate the performance of the WSN enslaved to RF energy. Section 5 presents a conclusion in which the prospects for expansion are suggested.

## 2. Previous Works and Contributions

**Figure 1:**
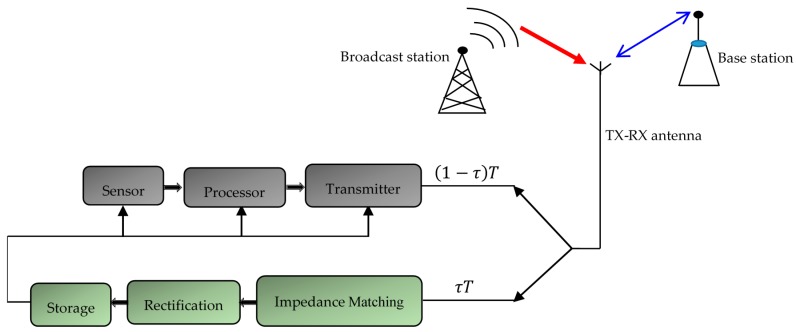
Conceptual view of enslaved sensor node.

In general, to harvest RF energy, an antenna is combined with an RF/DC converter and a high frequency filter that ensures the impedance matching between the antenna and the converter is sufficient [11]. This set constitutes a rectenna (Rectifying Antenna). Since the recovered energy is very small, it must be stored in a storage element (capacitor, battery, or supercapacitor). The block diagram of a rectenna is shown in the second part of Figure 1 [12]. The collection and transmission of environmental data (including temperature, pressure, humidity, light, etc.) pass through the steps shown in the first part of Figure 1. The sensor measures the physical phenomenon and transmits the data to the processor after having processed the data transfer via a transmitter to the Base Station (BS), or to another sensor node according to the configuration of the network [13].

The slave node consists of two parts: the node and the rectenna. Considering the principle of reciprocity of antennas [14], this work proposes to use the same antenna for transmitting data to the base station, and for recovering the surrounding ambient energy. This energy harvesting approach is known as the harvest-use (HU) architecture [8]. A conceptual view of the architecture suggested in this work is shown in Figure 1. The time switching approach is used and for each packet transmission, 0 <τ <1 is the fraction of time during which the energy is harvested; it corresponds to the sleep time of the sensor node, and (1−τ) is the fraction of time dedicated to the collection and transmission of the information. T is the total duration of a round. We assume that the antenna receives an incident power of 0 dBm during the energy harvest process, and that the losses in the polarization network and the radiating elements are negligible.

To achieve the objectives of this work, quantification of the energy needed by a sensor node in a WSN organized according to a protocol adapted to the RF source is proposed. It will then issue a new methodology for designing RF energy harvesters. Thus, the implementation of the slave node requires taking into account the improvements made in the field of network protocols, and advances made in the field of rectenna design.

### 2.1. WSN Topology Selection and Contribution

**Figure 2:**
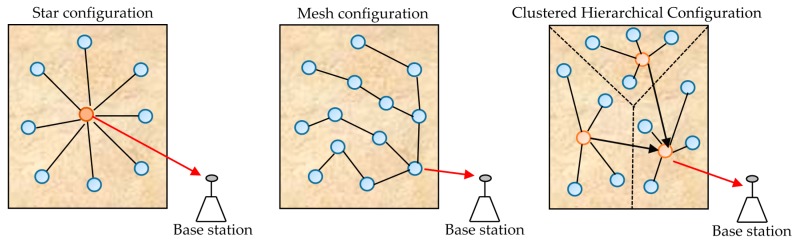
Main topologies of Wireless Sensors Network (WSN).

In the field of network protocols, several criteria are defined in the literature to characterize a WSN. Parameters such as reliability, data latency, energy efficiency, and network lifetime are often considered to compare the performance of WSNs [13]. However, in most cases, the sensor nodes must be deployed on large areas very often difficult to access; this requires designers to offer systems capable of running for as long as possible. That is why in the most recent works, energy efficiency and network lifetime of the node have been the two most important parameters in the design of WSNs [15]. With the advent of energy harvesting techniques, the issue of network lifetime can be solved by enslaving WSN performance to the amount of available ambient energy; this is the solution proposed in this work. This solution is motivated by recent research aimed at minimizing the energy requirement of wireless sensor nodes [16,17,18]. Note that the consumption of a sensor node depends on its function in the WSN, which is itself related to the topology of the network. Several topologies exist for WSNs; the main ones being, star, mesh and clustered networks [15] (Figure 2). 

In the star topology, all nodes transmit their data to a central node called sink or coordinator. The latter must, therefore, be provided with an amount of energy greater than that of the other nodes. In the mesh topology, some sensor nodes must, in addition to transmitting their own data, relay data from other nodes of the WSN. These sensor nodes are called gateway nodes, and therefore need more power than the other nodes of the WSN. In the cluster topology, before their transmission to the base station, the data are processed locally in regions called clusters; this contributes to distributing loads of the WSN between groups of sensor nodes arranged according to their positions in the WSN. In most research, it is the mesh topology that is considered, because it allows for the reduction of the transmission distance that helps to lessen the energy due to communication between different nodes [15,19].

Assuming that the controlled area is subject to the same amount of recoverable power, it is important to choose a topology that best distributes the WSN loads between the different nodes they contain. Thus, any optimization of the REHS (rectenna) helps to increase the capacity of the entire network. The cluster topology that distributes the WSN loads between the different clusters is the one that is best suited to our working hypothesis. The node’s consumption is also influenced by the algorithm that governs communications. Since it is inclined to an equitable distribution of network loads, the well-known Low-Energy Adaptive Clustering Hierarchy (LEACH) protocol is considered in this work [20]. Unlike previous work, several dissipation sources are taken into account to ensure an adequate design of the autonomous WSN because, in most previous works, only the energy dissipated due to the communication is considered [20,21,22]. However, other sources of dissipation, such as energy dissipated during the cluster-formation phase, energy for data acquisition, and transient energy, must be taken into account to define the WSN specifications. A comprehensive energy-consumption model has been proposed in [23]. However, the energy dissipated during cluster formation phase was not considered. The different sources of dissipation considered in this work are reported in Table 1; a comparison with the previous models is also shown.

### 2.2. Recent Progress in Rectenna Design and Contribution

Given the small amount of recoverable ambient RF energy, the main objective in rectenna design is to optimize the overall conversion efficiency of the system. The most common solution in the literature is to increase the power captured by the antenna. This results in the design of high-gain omnidirectional antennas [24] or the use of antenna arrays [25]. Other solutions considered by other researchers include designing matching filters to improve the RF/DC conversion efficiency of the system [11]. To increase the power and output voltage of the rectenna, interconnections of several rectennas known as rectenna arrays have also been proposed. In [26], nine rectennas were connected in series to power a mechanical actuator in a spatial application. The proposed circuit provided a DC voltage of 50 V. It should be noted that most of the above studies offer solutions that increase the size of the REHS, making it cumbersome for miniature applications, such as military applications where discretion is required. This work will focus on the loss due to electrical characteristics of the used rectifying diode. The small signal model of commonly used and marketed Schottky diodes will be considered and used to define the most appropriate diode for the study frequency band. Since most radio modules operate at 2.45 GHz [10], a comparison of the performance of the various commonly used diodes is made to minimize losses that occur during RF/DC conversion. Accordingly, this work discusses the development of a miniature high-efficiency rectifier, optimized for RF energy harvesting in the Industrial Scientific and Medical (ISM) band centralized at 2.45 GHz (Section 4).

## 3. A Comprehensive Energetic Budget of a Sensor Node in LEACH Clustering WSN

The LEACH protocol for WSN allows all data from nodes within the cluster to be processed locally, reducing the set of data that needs to be transmitted to the end user [27]. This idea was first proposed in [28], and the basic concept was to transmit data from sensor nodes, through its cluster head (CH), to the BS in rounds, and for each round, there is a permutation of the CH nodes to prevent their dying quickly. Each round consists of two phases: the set-up phase and the steady phase. In the set-up phase, each node decides whether or not to become a cluster head for the current round. In the steady phase, the CH nodes collect data from sensor nodes, aggregate them and send them to the BS. To provide a comprehensive energy model of the node, it is important to take into account all the operating phases of the sensor nodes in a LEACH WSN. However, in the model proposed in [20], which is the most frequently cited in the literature, the energy consumed during the set-up phase was neglected. This model only considers energy for microcontroller processing and energy for radio transmission and receiving data. In order to feed the sensor node with the ambient RF energy, it is important to provide an accurate energy consumption model of the node. In this work, the total energy consumed by the node is expressed as the sum of the energy dissipated during the steady phase and the set-up phase.

### 3.1. Dissipated Energy during the Set-Up Phase

During the set-up phase, the WSN is divided into clusters, and the different CH nodes are elected. The decision is based on a predetermined fraction of nodes, and a threshold T(n) which is set through this formula.

(1) $$T\left(n\right)=\{\begin{array}{c} \frac{p}{1-p*\left(r\;\mathrm{mod}\;\frac{1}{p}\right)\;}\;if\;n\in G\\ \;0\;\mathrm{otherwise} \end{array}$$

where G is the set of nodes that have not been CH in the previous 1/p rounds, r is the current round, and p is the predetermined percentage of clusters. Nodes that were not CH in previous 1/p rounds generate a number between 0 and 1; if less than the threshold T(n), then nodes become CH. The elected CH broadcasts its status using CSMA MAC protocol. Thus, all Cluster Member (CM) nodes must keep their receivers on during the set-up phase to hear the advertisements sent by the CH node. 

**Figure 3:**
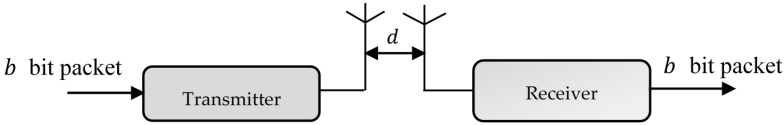
Classical radio channel model.

To define a sensor node’s energy requirement, this paper follows the radio model discussed in [28] described in Figure 3. The following reasonable assumptions are made:

- The Friis space model is considered inside the cluster, while the multipath fading model will be used for communication between the CH and the base station [29].
- All sensor nodes are homogeneous; the sensor nodes measure the same amount of data and are all located at an average distance $d_{1}$ from the CH.
- The fixed BS is located far from the sensor field; thus, all CHs are approximately at the same distance $d_{2}$ from the BS [20].
- The WSN includes N uniformly distributed sensors in an area of $M^{2}$; each cluster is circular and includes N/k nodes on a surface of $M^{2}/k$ [20].
- Single-hop transmission to the sink is assumed [23].
- CH is at the center of mass of its cluster [20,28].
- All sensor nodes within a cluster use time division multiple access (TDMA) to access their CH [30].

According to the model shown in Figure 3, the energy dissipated during the transfer of b bits of data between a transmitter and receiver node separated by a distance d is the sum of the energy consumed during the data transmission $E_{tx}$, the energy dissipated during the data reception $E_{rx}$, and the energy dissipated during the idle listening state $E_{lx}$. The energy consumed for data transmission is defined as [29]

(2) $$E_{tx}=\{\begin{array}{c} b\left(E_{\mathrm{elec}}+\varepsilon_{fs}.d_{1}^{2}\right)\;\mathrm{for}\;\mathrm{transmission}\;\mathrm{inside}\;\mathrm{the}\;\mathrm{cluster}\\ b\left(E_{\mathrm{elec}}+\varepsilon_{amp}.d_{2}^{4}\right)\;\mathrm{for}\;\mathrm{transmission}\;\mathrm{between}\;\mathrm{CH}\;\mathrm{and}\;\mathrm{BS} \end{array}$$

with

(3) $$d_{2}\geq \sqrt{\frac{\varepsilon_{fs}}{\varepsilon_{amp}}}$$

$E_{\mathrm{elec}}$ is energy consumption per bit in the transmitter and receiver circuitry, $\varepsilon_{fs}$ is the multiple attenuation model amplifier energy consumption, and $\varepsilon_{amp}$ is the transmit amplifier for two rays.

The energy consumed during data reception is given by

(4) $$E_{rx}=bE_{\mathrm{elec}}$$

Finally, the energy consumed in idle listening state is defined as

(5) $$E_{rx}=\beta bE_{\mathrm{elec}}$$

β is the ratio of reception and idle listening energy; it is taken as 0.85, since at the idle listening state, the radio dissipates 50% to 100% of the energy consumed in the receiving mode [31].

#### 3.1.1. CH Node Energy Consumption

During the set-up phase, all CH nodes send their location and approval request to the BS in CSMA mode. The CH nodes remain in idle listening mode, then advertise their status to all CM nodes [30]. Consequently, the energy dissipated by a CH node during the set-up phase can be defined as

(6) $$E_{{\mathrm{CH}}_{\mathrm{set}-\mathrm{up}\;\mathrm{phase}}}=\underset{\mathrm{send}\;\mathrm{location}\;\mathrm{to}\;\mathrm{BS}}{\underbrace{\frac{b_{1}}{\alpha }\left(E_{\mathrm{elec}}+\varepsilon_{amp}d_{2}^{4}\right)}}+\underset{\mathrm{Idle}-\mathrm{listening}}{\underbrace{\frac{b_{1}}{\alpha }E_{\mathrm{elec}}\beta }}+\underset{\mathrm{advertise}\;\mathrm{of}\;\mathrm{status}}{\underbrace{b_{1}\left(E_{\mathrm{elec}}+\varepsilon_{fs}d_{1}^{2}\right)}}$$

where $b_{1}$ is the control packet [32] size, and α is the throughput of non-persistent CSMA; it is defined in [33] as

(7) $$\alpha =\frac{b_{1}e^{\left(-ab_{1}\right)}}{\left(1+2a\right)b_{1}+e^{\left(-ab_{1}\right)}}$$

where a is the ratio of propagation delay to packet transmission time. For control packet size $b_{1}=200\;\mathrm{bits}$ [33], and taking a=0.01, the value of α comes out to be 0.132. 

Since the CH is the center of mass of the cluster, the expected squared distance from the nodes to the cluster head $d_{1}^{2}$ is defined as

(8) $$E\left[d_{1}^{2}\right]=\iint d\left(x,y\right)\rho \left(x,y\right)dxdy=\iint r^{2}\rho \left(r,\theta \right)rdrd\theta$$

where ρ(r,θ) is the joint probability density function. If the sensor nodes are distributed uniformly,$\;\rho \left(r,\theta \right)=\rho \left(x,y\right)=k/M^{2}$. In the case of a circular surface as in [20], the mean square distance from a CM to its CH is given by

(9) $$E\left[d_{1}^{2}\right]=\frac{k}{M^{2}}\overset{2\pi }{\underset{0}{\int }}\left(\overset{M/\sqrt{\pi k}}{\underset{0}{\int }}r^{3}dr\right)d\theta =\frac{M^{2}}{2\pi k}$$

Substituting Equation (9) into Equation (6) gives

(10) $$E_{{\mathrm{CH}}_{\mathrm{set}-\mathrm{up}\;\mathrm{phase}}}=\frac{b_{1}}{\alpha }\left(E_{\mathrm{elec}}+\varepsilon_{amp}d_{2}^{4}\right)+\frac{b_{1}}{\alpha }E_{\mathrm{elec}}\beta +b_{1}\left(E_{\mathrm{elec}}+\varepsilon_{fs}\frac{M^{2}}{2\pi k}\right)$$

#### 3.1.2. CM Node Energy Consumption

Once the CH node is elected, it broadcasts to advertise its CH election using the CSMA MAC protocol. Thus, all CM nodes must keep their receivers on during the set-up phase to hear the advertisements sent by the CH node [30]. The dissipated energy by a CM node during the set-up phase is then expressed as

(11) $$E_{{\mathrm{CM}}_{\mathrm{set}-\mathrm{up}\;\mathrm{phase}}}=\frac{b_{1}}{\alpha }E_{\mathrm{elec}}\beta$$

### 3.2. Dissipated Energy during the Steady Phase

**Figure 4:**
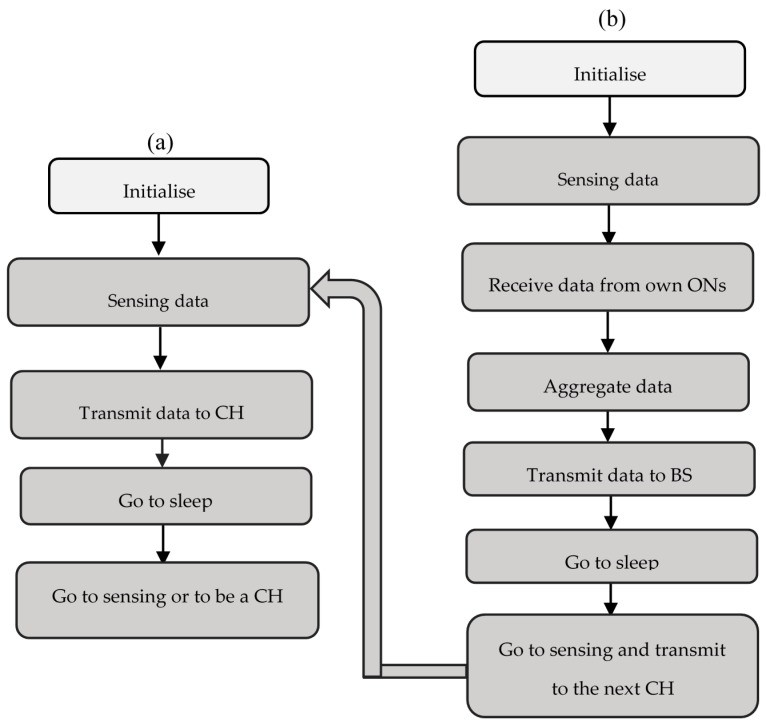
(**a**) CM behavior during the steady phase (**b**) CH behavior during the steady phase.

Once the network is divided into clusters, a CH node computes a TDMA schedule for its CM nodes, specifying when the CM node in the cluster is allowed to send its data. The CM node has to sense data and transmit to CH; while the CH node has to collect data from CM nodes, aggregate them, and send it to the BS. The behavior of the two types of nodes is illustrated by diagrams shown in Figure 4.

#### 3.2.1. CM Node Energy Consumption during the Steady Phase

The sensing system links the sensor node to the physical world. The total energy dissipation for data acquisition $E_{\mathrm{acqui}\;}$ of b bits packet is expressed in [13,24] as

(12) $$E_{{\mathrm{acqui}}_{\mathrm{CM}}\;}=\underset{\mathrm{data}\;\mathrm{capture}}{\underbrace{bV_{\sup}I_{\mathrm{sens}}T_{\mathrm{sens}}}}\;+\underset{\mathrm{data}\;\mathrm{recording}\;}{\underbrace{\frac{bV_{\sup}}{8}\left(I_{\mathrm{read}}T_{\mathrm{read}}+I_{\mathrm{write}}T_{\mathrm{write}}\right)}}$$

where $V_{\sup}$ is the supply voltage. $I_{\mathrm{sens}}$ and $T_{\mathrm{sens}}$ are, respectively, the total current required for sensing activity the time duration for sensor node sensing. $I_{\mathrm{read}}$ and $I_{\mathrm{write}}$ are, respectively, the current for reading 1-byte data and current for writing 1-byte data. $T_{\mathrm{read}}$ and $T_{\mathrm{write}}$ are, respectively, the time duration for reading 1-byte data and time duration for writing 1-byte data.

Once it has collected measures, the CM node just has to transmit them to the CH, as shown in Figure 4. According Equation (2) and considering Equation (9), the corresponding dissipated energy is expressed as

(13) $$E_{tx_{\mathrm{CM}}}=b\left(E_{\mathrm{elec}}+\varepsilon_{fs}\frac{M^{2}}{2\pi k}\right)$$

During a round, the sensor node must switch between the active, idle, and sleep mode to save energy. This change in radio operating mode can cause a significant amount of power dissipation. The dissipated energy due to this state change is defined in [34] as

(14) $$E_{{\mathrm{trans}}_{\mathrm{CM}}}=T_{A_{\mathrm{CM}}}V_{\sup}\left[\alpha_{\mathrm{CM}}I_{A}+\left(1-\alpha_{\mathrm{CM}}\right)I_{S}\right]$$

where $T_{A_{\mathrm{CM}}}$ is the active time of the CM node, $I_{A}$ and $I_{S}$ are, respectively, the current consumed during the wake-up mode and current consumed during the sleeping mode, and $\alpha_{\mathrm{CM}}$ is the duty cycle of the CM node, defined in [34] as

(15) $$\alpha_{\mathrm{CM}}=\frac{T_{\mathrm{transON}}+T_{A_{\mathrm{CM}}}+T_{\mathrm{transOFF}}}{T_{\mathrm{transON}}+T_{A_{\mathrm{CM}}}+T_{\mathrm{transOFF}}+T_{S_{\mathrm{CM}}}}$$

where $T_{S_{\mathrm{CM}}}$ is the sleep time, $T_{\mathrm{transON}}$ and $T_{\mathrm{transOFF}}$ are, respectively, the time required for sleep-to-idle and idle-to-sleep transitions.

The total energy dissipated by a CM node during the steady phase is then defined according to the diagram of Figure 4 as

(16) $$E_{{\mathrm{CM}}_{\mathrm{steady}\;\mathrm{phase}}}=E_{{\mathrm{acqui}}_{\mathrm{CM}}}+\;E_{tx_{\mathrm{CM}}}+E_{{\mathrm{trans}}_{\mathrm{CM}}}$$

#### 3.2.2. CH Node Energy Consumption

The CH node has to capture data, record them, collect data from CM nodes, aggregate them, and send them to the BS. The energy dissipated for the data acquisition is the same as that of a CM node.

(17) $$E_{{\mathrm{acqui}}_{\mathrm{CH}}\;}=\underset{\mathrm{Sensing}}{\underbrace{bV_{\sup}I_{\mathrm{sens}}T_{\mathrm{sens}}}}+\;\underset{\mathrm{Data}\;\mathrm{recording}}{\underbrace{\frac{bV_{\sup}}{8}\left(I_{\mathrm{read}}T_{\mathrm{read}}+I_{\mathrm{write}}T_{\mathrm{write}}\right)}}$$

The total energy dissipation by the CH node used for data aggregation of bN/k bits, $E_{{\mathrm{mic}}_{\mathrm{CH}}}$ per round is given by [23,35]

(18) $$E_{{\mathrm{mic}}_{\mathrm{CH}}}=\frac{bN}{k}V_{\sup}N_{\mathrm{cyc}}\left(\frac{I_{0}}{f}e^{\frac{V_{\sup}}{n_{p}V_{t}}}+C_{\mathrm{avg}}V_{\sup}\right)$$

$N_{\mathrm{cyc}}$ is the number of clock cycles per task, $I_{0}$ is the leakage current, f is the sensor frequency, $n_{p}$ is a constant which depends on the processor, $V_{t}$ is the thermal voltage, and $C_{\mathrm{avg}}$ is the average capacitance switched per cycle.

According to Equation (2), energy dissipation due to the transmission of bN/k bits of data over a distance $d_{2}$ is defined as

(19) $$E_{tx_{\mathrm{CH}}}=\frac{bN}{k}\left(E_{\mathrm{elec}}+\varepsilon_{amp}d_{2}^{4}\right)$$

Similarly to the CM node, the transient energy in the CH node is defined as

(20) $$E_{{\mathrm{trans}}_{\mathrm{CH}}}=T_{A_{\mathrm{CH}}}V_{\sup}\left[\alpha_{\mathrm{CH}}I_{A}+\left(1-\alpha_{\mathrm{CH}}\right)I_{S}\right]$$

where $T_{A_{\mathrm{CH}}}$ is the active time of the CH node given by

(21) $$T_{A_{\mathrm{CH}}}=\left(\frac{N}{k}-1\right)T_{A_{\mathrm{CM}}}+T_{\mathrm{CH}-\mathrm{sink}}$$

with $T_{\mathrm{CH}-\mathrm{sink}}$ which represents the time to transmit data to BS. The duty cycle of the CH node $\alpha_{\mathrm{CH}}$ is then defined as

(22) $$\alpha_{\mathrm{CH}}=\frac{T_{\mathrm{transON}}+T_{A_{\mathrm{CH}}}+T_{\mathrm{transOFF}}}{T_{\mathrm{transON}}+T_{A_{\mathrm{CH}}}+T_{\mathrm{transOFF}}+T_{S_{\mathrm{CH}}}}$$

where $T_{S_{\mathrm{CH}}}$ is the sleep time of the CH node defined as

(23) $$T_{S_{\mathrm{CH}}}=T-T_{A_{\mathrm{CH}}}=\tau T$$

with T being the duration of a round.

The total energy consumption of the CH node per round during the steady phase can now be calculated using Equation (24).

(24) $$E_{{\mathrm{CH}}_{\mathrm{steady}\;\mathrm{phase}}}=E_{{\mathrm{aqui}}_{\mathrm{CH}}}\left(b\right)+E_{{\mathrm{mic}}_{\mathrm{CH}}}\;\left(b\right)+\;E_{tx_{\mathrm{CH}}}\left(b,d_{2}\right)+E_{{\mathrm{trans}}_{\mathrm{CH}}}$$

### 3.3. Optimal Number of Cluster and Node Energy Consumption

From the above, the total energies consumed by the CM node $E_{\mathrm{CM}}$ and the CH node $E_{\mathrm{CH}}$ per round are given by Equation (25).

(25) $$\{\begin{array}{c} E_{\mathrm{CM}}=E_{{\mathrm{CM}}_{\mathrm{set}-\mathrm{up}\;\mathrm{phase}}}+\;E_{{\mathrm{CM}}_{\mathrm{steady}\;\mathrm{phase}}}\\ E_{\mathrm{CH}}=E_{{\mathrm{CH}}_{\mathrm{steady}\;\mathrm{phase}}}+\;E_{{\mathrm{CH}}_{\mathrm{steady}\;\mathrm{phase}}} \end{array}$$

The energy dissipated in a cluster per round can be defined as

(26) $$E_{\mathrm{cluster}}=\left(\frac{N}{k}-1\right)E_{\mathrm{CM}}+E_{\mathrm{CH}}$$

and the total energy per round is

(27) $$E_{\mathrm{WSN}}=kE_{\mathrm{cluster}}=\left(N-k\right)E_{\mathrm{CM}}+kE_{\mathrm{CH}}$$

By substituting Equations (10), (11), (16), and (24) into Equation (25), and the obtained result into Equation (27), the total energy dissipated by the WSN is expressed as

(28) $$\begin{array}{cc} E_{\mathrm{WSN}}=A+ℬk & +C\left(kE_{\mathrm{elec}}+\varepsilon_{fs}\frac{M^{2}}{2\pi }\right)+\left(k\frac{b_{1}}{\alpha }+bN\right)\left(E_{\mathrm{elec}}+\varepsilon_{amp}d_{2}^{4}\right)\\ & +bNV_{\sup}N_{\mathrm{cyc}}\left(\frac{I_{0}}{f}e^{\frac{V_{\sup}}{n_{p}V_{t}}}+C_{\mathrm{avg}}V_{\sup}\right)+N\;b\left(E_{\mathrm{elec}}+\varepsilon_{fs}\frac{M^{2}}{2\pi k}\right) \end{array}$$

with A, ℬ and C defined as in Equation (29):

(29) $$\{\begin{array}{c} A=N\frac{b_{1}}{\alpha }E_{\mathrm{elec}}\beta +NE_{{\mathrm{acqui}}_{\mathrm{CM}}}+NE_{{\mathrm{trans}}_{\mathrm{CM}}}\;\\ B=E_{{\mathrm{aqui}}_{\mathrm{CH}}}\;+E_{{\mathrm{trans}}_{\mathrm{CH}}}-E_{{\mathrm{acqui}}_{\mathrm{CM}}}-E_{{\mathrm{trans}}_{\mathrm{CM}}}=E_{{\mathrm{trans}}_{\mathrm{CH}}}-E_{{\mathrm{trans}}_{\mathrm{CM}}}\\ C=b_{1}-b\; \end{array}$$

The optimal number of clusters is determined by setting the derivative of $E_{\mathrm{WSN}}$ with respect to k to zero. This gives Equation (30).

(30) $$k_{\mathrm{opt}}=M\frac{\sqrt{N}}{\sqrt{2\pi }}\sqrt{\frac{b\;\varepsilon_{fs}}{ℬ+CE_{\mathrm{elec}}+\frac{b_{1}}{\alpha }\left(E_{\mathrm{elec}}+\varepsilon_{amp}d_{2}^{4}\right)}}$$

**Figure 5:**
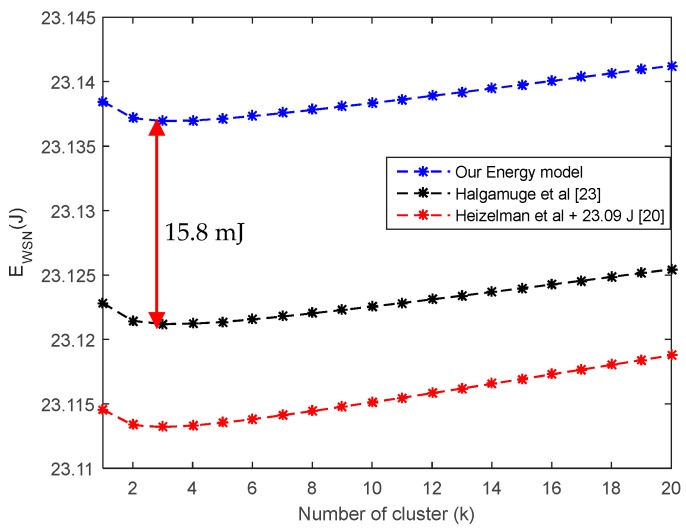
Average energy dissipation versus number of clusters $d_{\mathrm{CH}-\mathrm{BS}}=110\;m$.

Considering Equation (29), a representation of the total energy dissipated in the WSN based on the number of clusters is shown in Figure 5; the comparison with other energy models is also made. The parameters used for the comparison are reported in Table 2. The sleeping time and the current required for sensor wake-up are those of Mica2 Motes [36]. The radio parameters such as sensor wake-up and sleeping time used here are those of CC2520 of Texas Instruments [37]. A difference of 15.8 mJ is observed between our energy model and that proposed in [23]. This difference is considerable given the small amount of recoverable ambient energy [38].

In the model defined in [20], only the energies dissipated due to the communication and to the processing of the data were considered. The total energy consumed by the network has been defined as

(31) $$E_{\mathrm{WSN}-\mathrm{Hein}}=b\left(2NE_{\mathrm{elec}}+NE_{{\mathrm{mic}}_{\mathrm{CH}}}+k\varepsilon_{amp}d_{2}^{4}+\varepsilon_{fs}\frac{N}{2\pi }\frac{M^{2}}{k}\right)$$

The optimal number of clusters in this case was defined as

(32) $$k_{{\mathrm{opt}}_{\mathrm{Hein}}}=\frac{\sqrt{N}}{\sqrt{2\pi }}\sqrt{\frac{\varepsilon_{fs}}{\varepsilon_{amp}}}\frac{M}{d_{2}^{2}}$$

According to [23], the total energy during each round $E_{\mathrm{WSN}-\mathrm{Halg}}$ is given by

(33) $$E_{\mathrm{WSN}-\mathrm{Halg}}=b(2NE_{\mathrm{elec}}+NE_{{\mathrm{mic}}_{\mathrm{CH}}}+d_{2}^{4}\varepsilon_{amp}k+E_{{\mathrm{acqui}}_{\mathrm{CH}}\;}k+E_{{\mathrm{trans}}_{\mathrm{CH}}}k+\varepsilon_{fs}\frac{M^{2}}{k}N\;+E_{{\mathrm{trans}}_{\mathrm{CM}}}N+E_{{\mathrm{acqui}}_{\mathrm{CM}}}N)$$

with the optimal number of square cluster defined as

(34) $$k_{{\mathrm{opt}}_{\mathrm{Halga}}}=\frac{\sqrt{N}}{\sqrt{6}}\frac{M}{d_{2}^{2}}\sqrt{\frac{\varepsilon_{fs}}{\varepsilon_{amp}+E_{{\mathrm{acqui}}_{\mathrm{CH}}\;}+E_{{\mathrm{trans}}_{\mathrm{CH}}}}}$$

**Figure 6:**
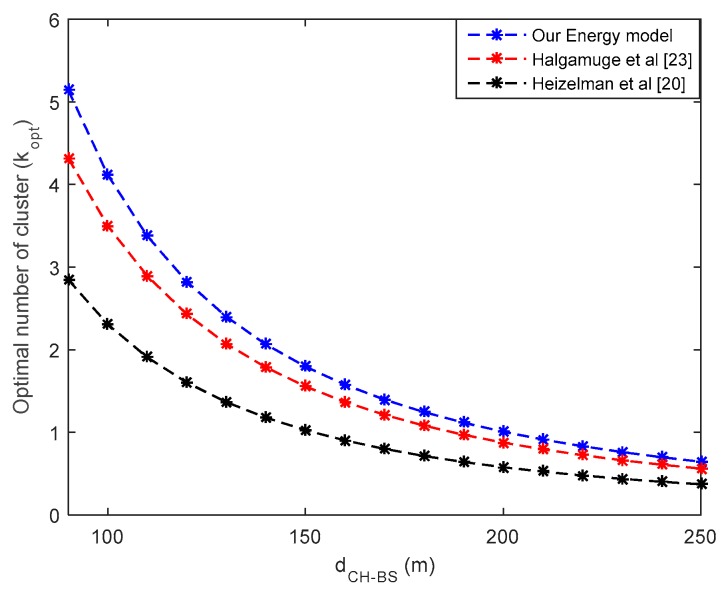
Optimal number versus distance from CH to BS.

In Figure 6, it can be seen that the optimum number of clusters for the same number of wireless nodes in the network is larger in our energy model. This contributes to an increase in inter-cluster interference, because the more clusters there are, the greater the likelihood that simultaneous communications will increase the risk of interference in the network; this issue is not addressed in this work. It is important to note that the optimum cluster numbers get closer as the range of the WSN increases. This validates the result of Figure 5, in which the optimal cluster number is almost the same in the three models compared in this work.

**Figure 7:**
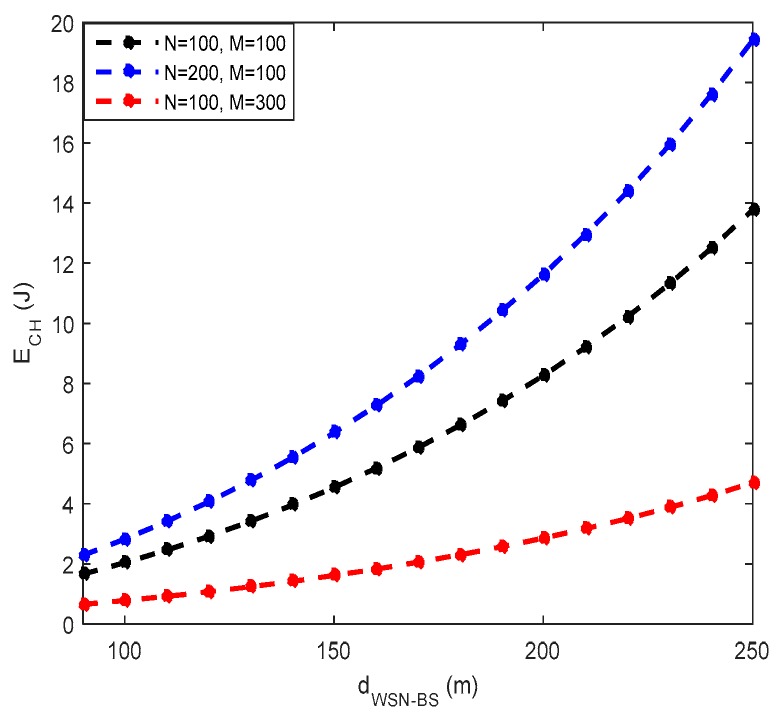
Consumption of the CH node as a function of WSN–BS station distance.

**Figure 8:**
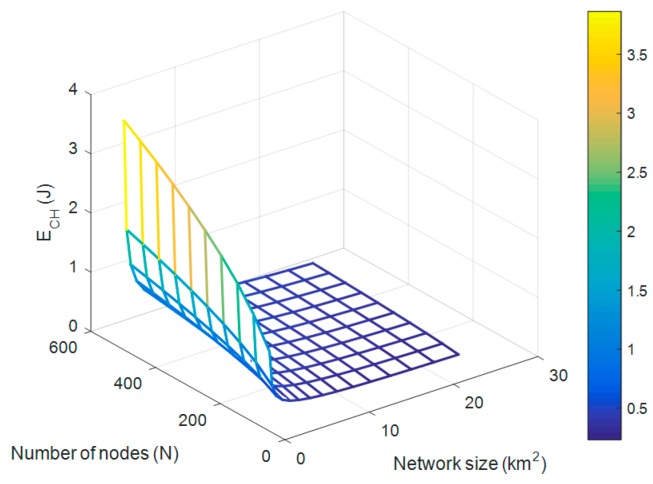
CH node energy dissipation versus the number of nodes and the network size $d_{\mathrm{WSN}-\mathrm{BS}}=150\;m$.

The operating condition of the autonomous WSN requires that the amount of recovered RF energy is greater than the energy requirement of a CH node. A representation of the energy dissipated by the CH node during a round as a function of the distance separating the WSN from the BS is thus represented in Figure 7. It appears that for a WSN with 100 nodes uniformly distributed over a surface of $100\;\times \;100\;m^{2}$, the CH node needs an amount of energy that can range from 0.65 J to 4.6 J  for a WSN–BS distance varying between 90 and 250 m. If the number of nodes is increased, this increases the energy consumption of CH, because the number of data to be received and processed by the CH node increases. For a larger area to be controlled, the energy requirement of CH is lower. According to Figure 7, it is then deduced that the consumption of the CH node decreases with the increase of the size of the WSN, and increases with the increase of the number of nodes. This general result is shown in Figure 8. In the continuation of this work, the $d_{\mathrm{CH}-\mathrm{BS}},\;\;M$ and N parameters will be used to enslave the sensor node to a rectenna designed to achieve optimal performance in the 2.45 GHz frequency band.

## 4. High Efficiency Rectifier Design for RF Energy Harvesting at 2.45 GHz

Most of the radio modules used in sensor nodes operate at a frequency of 2.45 GHz  [10]. In the case where the same antenna is used for transmitting data and retrieving the ambient energy, this section discusses the design of a rectenna with optimal operation in the ISM band centralized at 2.45 GHz. Given the random input RF power, it is important to optimize the harvesting circuit to have a minimum of usable energy. In this work, optimization will be to design a highly efficient rectifier circuit. The proposed circuit is based on a judicious choice of rectifier diode, which besides having good conversion efficiency, must be highly sensitive to detect low levels of available power. The output characteristics of the designed rectenna will then be used to evaluate the performance of the slave node.

### 4.1. Rectifier Diode Selection

**Figure 9:**
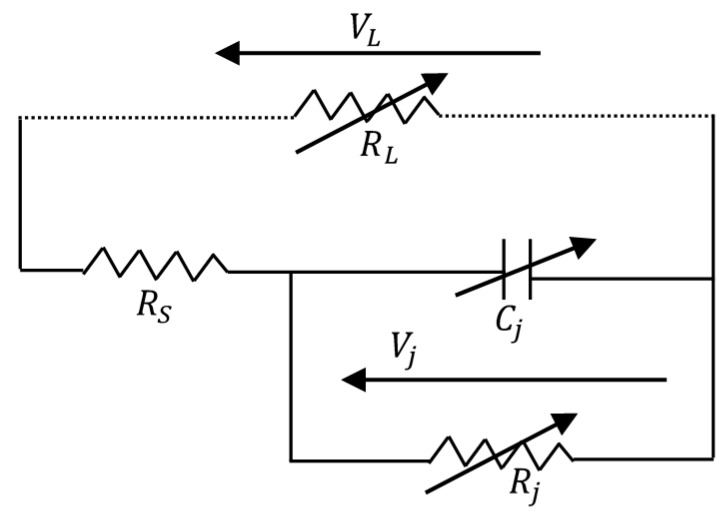
Small signal model of a Schottky diode [40,41].

The RF/DC converter in a rectenna allows for the conversion of RF power captured by the antenna into electrical DC power. Given the very low received power density [38], it is important to design a high-sensitivity rectifier circuit to have an acceptable amount of usable DC power. The sensitivity of the rectifier is directly related to the sensitivity of the used diode. The influencing factor on rectenna efficiency is the diode efficiency, and a significant portion of the losses on rectenna circuit is provided by the diode’s electrical parameters. Considering the high frequency of the signals, fast-switching Schottky diodes are the most frequently used in the design of rectifier circuits. To evaluate the conversion efficiency of a Schottky diode, the equivalent circuit of the diode with a resistive load $R_{L}$ proposed in [40], and shown in Figure 9 is used.

In Figure 9, $R_{S}$ is the series resistance; $C_{J}$ is the junction capacitance, $V_{j}$ the voltage across the semiconductor–metal junction, and $R_{j}$ the junction resistance. The most commonly used diodes are manufactured by Avago [41]. A non-exhaustive list of recently used rectifying diodes is given in Table 3. The electrical characteristics provided by the diode datasheets [42,43,44,45] are also reported. 

The diode RF/DC efficiency is expressed as a function of the load resistance $R_{L}$, internal elements of the diode ($R_{S}$,$\;C_{J}$,$\;R_{j}$,$\;V_{j}$) and the signal frequency ω (ω=2πf); it is defined as

(35) $$\eta_{\mathrm{RF}-\mathrm{DC}}=\frac{P_{DC}}{P_{RF}}$$

where $P_{DC}$ is the measured output DC power on the load resistance, and $P_{RF}$ the measured RF power delivered to the diode. In [40], the RF/DC conversion efficiency has been expressed regarding the internal parameters of the diode as

(36) $$\eta_{\mathrm{RF}-\mathrm{DC}}=\frac{1}{1+X+Y+Z}$$

with

(37) $$\{\begin{array}{c} X=\frac{R_{L}}{\pi R_{S}}{\left(1+\frac{V_{j}}{V_{L}}\right)}^{2}\left[\theta \left(1+\frac{1}{2\;\cos^{2}\theta }\right)-1,5\tan\theta \right]\\ Y=\frac{R_{S}\cdot R_{L}\cdot C_{j}^{2}\cdot \omega^{2}}{2\pi }\left(1+\frac{V_{j}}{V_{L}}\right)\left[\frac{\pi -\theta }{\cos^{2}\theta }+\tan\theta \right]\;\\ \;Z=\frac{R_{L}}{\pi R_{S}}\left(1+\frac{V_{j}}{V_{L}}\right)\frac{V_{j}}{V_{L}}\left[\tan\theta -\theta \right]\; \end{array}$$

where $V_{L}$ is the output self-bias DC voltage across the resistive load. θ is the forward-bias turn-angle; it is a dynamic variable that depends on the input power of the diode (and thus of the output DC voltage) as follows [40]

(38) $$\tan\theta -\theta =\frac{\pi R_{S}}{R_{L}\left(1+\frac{V_{j}}{V_{L}}\right)}$$

The junction capacitance $C_{j}$ is a function of $C_{j0}$ as

(39) $$C_{j}=C_{j0}\sqrt{\frac{V_{j}}{V_{j}+V_{L}}}$$

**Figure 10:**
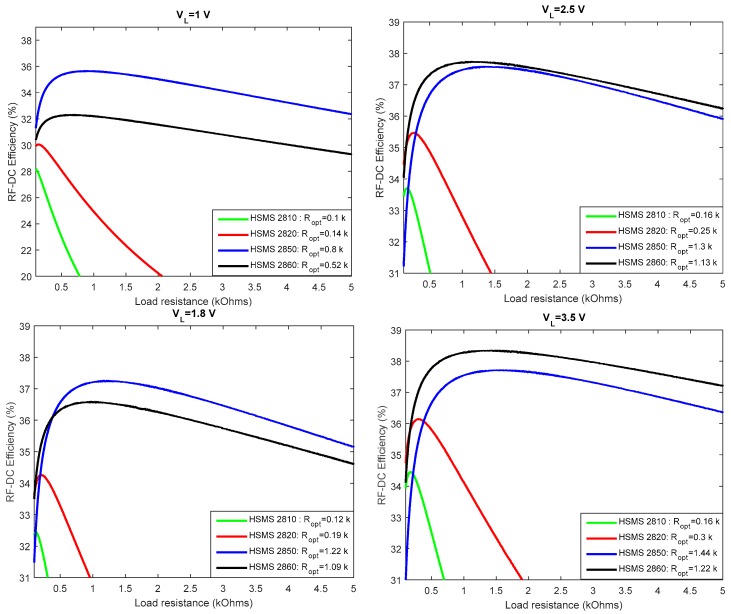
Efficiency as a function of load resistance for different levels of the output voltage.

Considering the diode’s characteristics given in Table 3, Equation (36) to Equation (39) are used to compare the efficiencies of the different diodes at 2.45 GHz. The result is shown in Figure 10, and it is observed that diodes HSMS 2850 and HSMS 2860 show the best efficiencies compared to the other considered diodes. However, at low output DC voltage, it is the HSMS 2850 diode that offers the best conversion efficiency. In this work, an output voltage of 1.8 V is required to supply the sensor, so diode HSMS 2850 will be used to design the rectifier circuit. Moreover, considering the previous work, it is difficult to reach output DC voltage levels of more than 2 V with a rectenna without exceeding the permissible levels of exposure.

**Figure 11:**
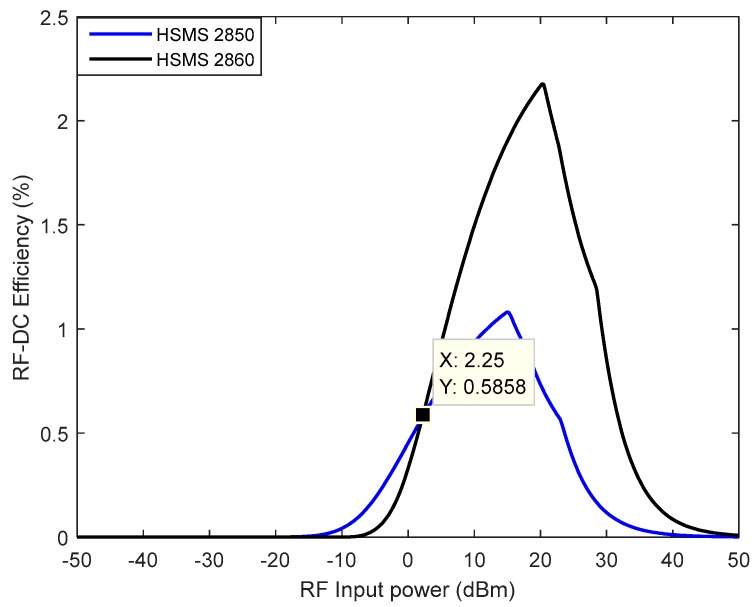
Comparison of the detection threshold of the diodes.

**Figure 12:**
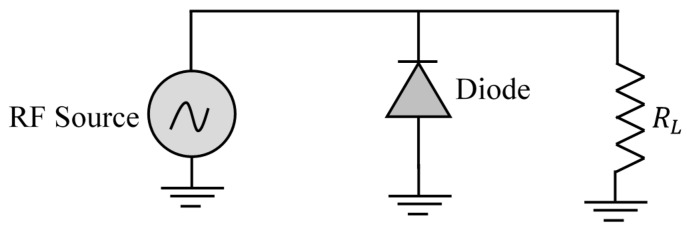
Schematic for the diode detection threshold.

A simulation of the detection threshold of the two diodes HSMS 2850 and HSMS 2860 is shown in Figure 11. The analysis is conducted using Advanced Design System (ADS) software, and the simulated circuit is that of Figure 12. For each diode, the value of the load resistance is set to that determined in Figure 10 when $V_{L}=1.8\;V$ (supply voltage to sensor).

Figure 11 validates the result in Figure 10 because it is shown that diode HSMS 2850 is the most efficient at low power levels (below 0.6 dBm).

### 4.2. Designed Rectifier and Measurements

**Figure 13:**
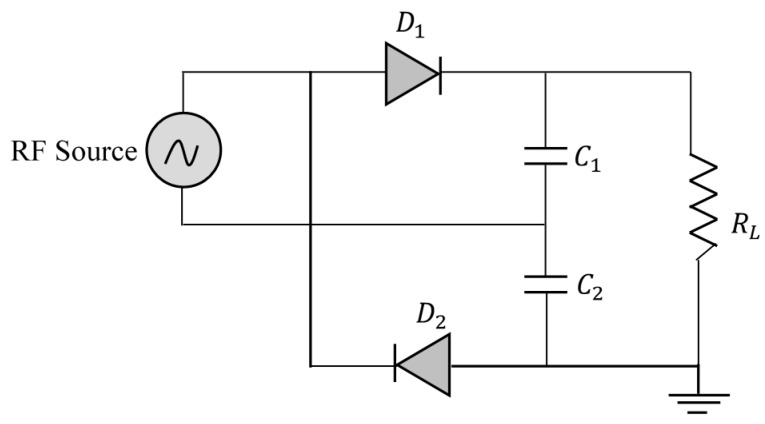
Used rectifier topology.

Four rectifier topologies are commonly used in rectenna design; namely, a single series diode, single parallel diode, the full bridge, and the Voltage Doubler (VD). A comparison of the performance of these different topologies has been proposed in [46], and for average power levels, it is the VD topology that has proven to be the most efficient. Several VD topologies exist, the Latour VD shown in Figure 13 is used in this work. If an alternating voltage $v\;=V_{\max}\sin\left(\omega t\right)$ is applied to the input of the circuit, diode $D_{1}$ is turned on during the positive half-wave while diode $D_{2}$ is cut-off. During this time, the capacitance $C_{1}$ is loaded to the value$\;V_{\max}$. At the negative half-cycle, diode $D_{2}$ is turned on and diode $D_{1}$ remains cut-off, the capacitance $C_{2}$ is loaded to the value $V_{\max}$, which gives a difference potential across the load of $2V_{\max}$.

**Figure 14:**
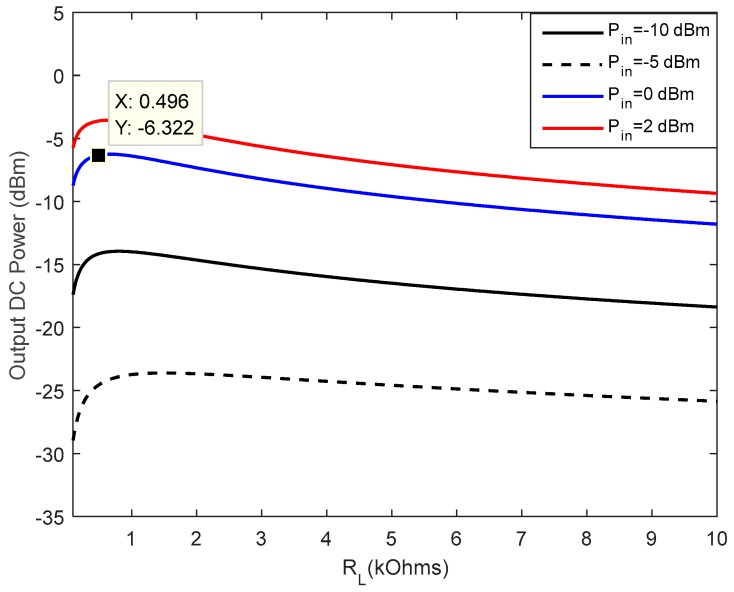
Optimal load resistance of the rectifier.

The first step in our design is to determine of the optimum load resistance of the circuit. The capacitors are set at 2.7 pF, and the optimum load resistance of the circuit is determined from the ADS software simulations. The output DC power is simulated with respect to the rectenna load resistance at different levels of input power (Figure 14). It is observed that the maximum power is obtained around 500 Ω when the input RF power is set at 0 dBm (1 mW).

**Figure 15:**
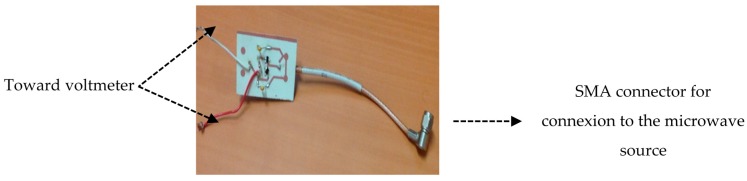
Fabricated Printed Circuit Board (PCB) of the Rectifier.

By setting load resistance of 470 Ω on the rectifier circuit, an experimental validation of the designed rectifier circuit is implemented. The simulated schematic and corresponding fabricated circuit are shown in Figure 15. To achieve the circuit, an RO350B substrate ($\varepsilon_{r}=3.4$, h=0.76 mm, T=35 μm,  tanδ=0.0037) from the Rogers Corporation, was chosen. A SubMiniature version A (SMA) connector is used to connect the rectifier to the microwave source.

**Figure 16:**
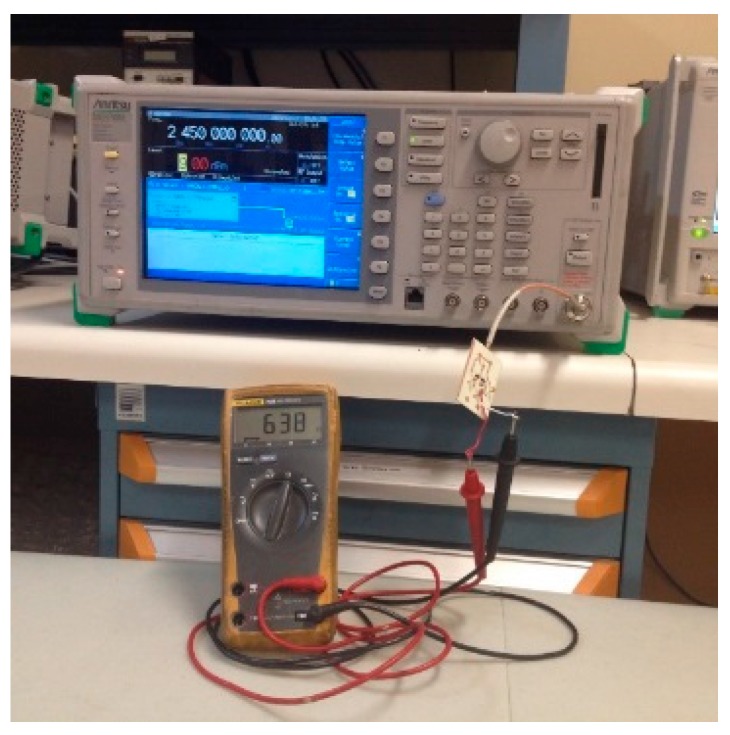
Experimental setup.

The experimental set-up shown in Figure 16 is used. It includes an Aritsu microwave source, MG3700A, with an internal impedance of 50 Ω that is able to transmit signals up to 6 GHz. The delivered output power reaches a maximum of 13 dBm and a minimum of −140 dBm.

**Figure 17:**
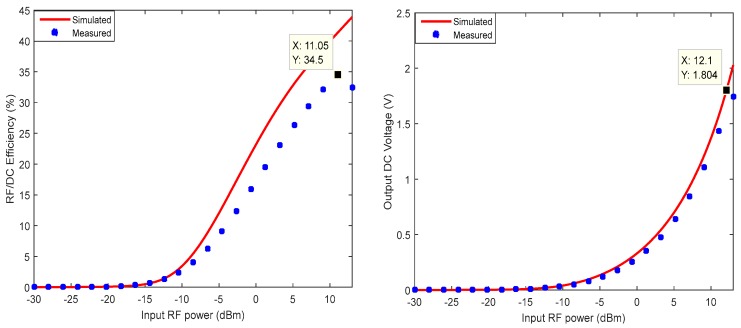
Rectifier measurements results.

The experimental results shown in Figure 17 are easily comparable to the simulated results. A slight distortion is observed on the experimental curve, due to the parasitic elements of the housing, which are not taken into account in the simulation. At 11.05 dBm of input RF power, a maximum conversion efficiency of 34.5% is achieved. An output voltage of 1.8 V can only be reached when the resulting circuit receives an input RF power of 12.1 dBm, which would be difficult to provide to the rectifier given the various attenuations (reflection, diffraction, and refraction) that the signal undergoes in a real environment [29].

Note that the performance shown in Figure 17 are achieved without the use of a matching filter between the microwave source and the rectifier circuit. In Table 4, a comparison of these performances with related design at 2.45 GHz is shown.

### 4.3. Rectifier Performance Improvement

**Figure 18:**
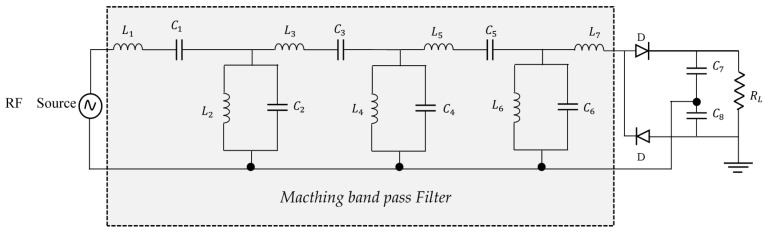
Rectifier with matching circuit.

A matching circuit must be placed between the microwave source and the rectifier to ensure optimum power transfer (Figure 1). In this work, the ADS impedance matching tool is used to size the filter. This tool allows us to place a component in our scheme, and according to certain parameters that are manually defined, generate a matching circuit that can then be optimized according to our goals. A bandpass filter is considered as in [49]. The schematic of the generated filter and rectifier circuit are shown in Figure 18. The optimization included in ADS software is used to find the best matching circuit and achieve better performance both in terms of efficiency and output voltage. The optimization used in this work refers to the gradient method search. This approach adjusts a set of variables according to an error function and its gradient. In the first iteration, the simulator evaluates the error function and its gradient. Subsequently, all variables are moved in the direction of the gradient of the error, thereby minimizing the error function [50]. The error function is the Least-Squares error function. After designing a matching circuit, all components are optimized by setting two goals at the same time

- minimizing return loss between 2.3 GHz and 2.6 GHz;
- maximizing the DC output voltage.

**Figure 19:**
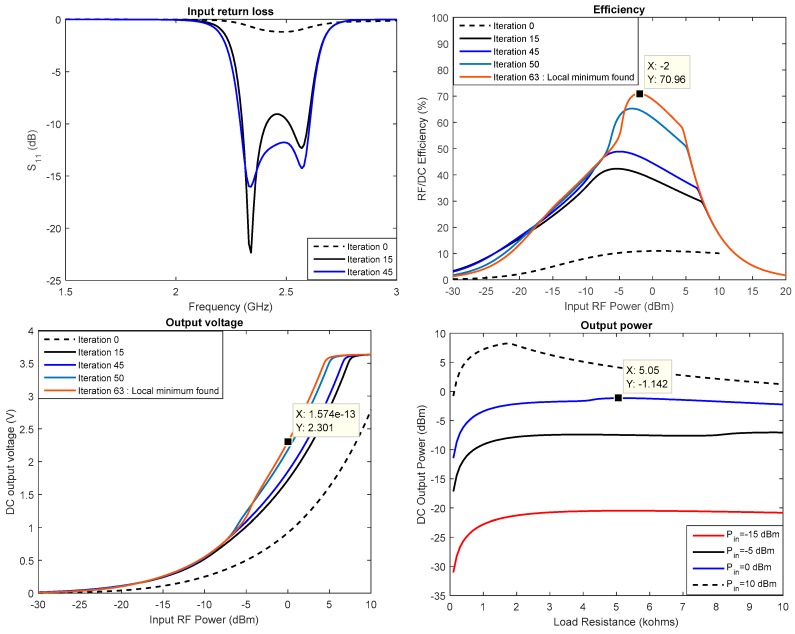
Optimized rectifier performance (simulated results).

The results obtained after 45 iterations are shown in Figure 19. For an input power of 0 dBm, a 2.3 V output voltage is reached. A maximum conversion efficiency of 71% is reached at around −2 dBm. The component values to achieve these performances are reported in Table 5.

To evaluate the contribution of the design methodology proposed here, Table 6 shows a comparison of the achieved performance with those obtained in recent designs at 2.45 GHz. It can be seen that our design offers high performance; this for an input power level lower than those obtained in the other circuits. This is an advantage for ambient energy harvesting applications because the RF energy naturally available in the environment is generally small [38].

It is also shown in Figure 19 that the optimal load changes with the RF input power. A Maximum Power Point Tracking (MPPT) block is necessary to keep track of the maximum efficiency operating condition. This issue is not discussed here, as MPPT is now a classical function. At 0 dBm of input power, a maximum of −1.42 dBm (0.77 mW) is reached for optimum load resistance of 5.05 kΩ. Assuming that all this recovered power is dedicated to operation of the sensor node, the available energy $\left(E_{\mathrm{rec}}\right)$ during time τT is defined by

(40) $$E_{\mathrm{rec}}=P_{\max}\tau T$$

where $P_{\max}$ is the maximum recoverable power. The sensor node can operate only if ($E_{\mathrm{rec}}\geq E_{\mathrm{CH}}$).

**Figure 20:**
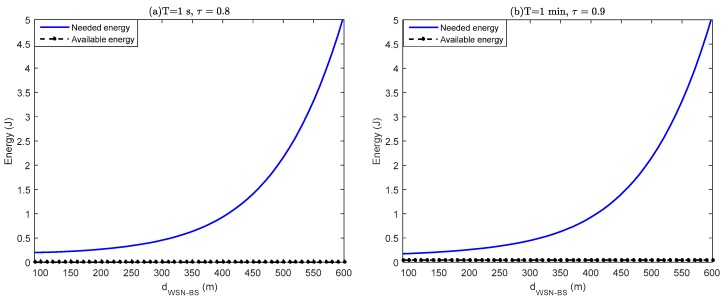

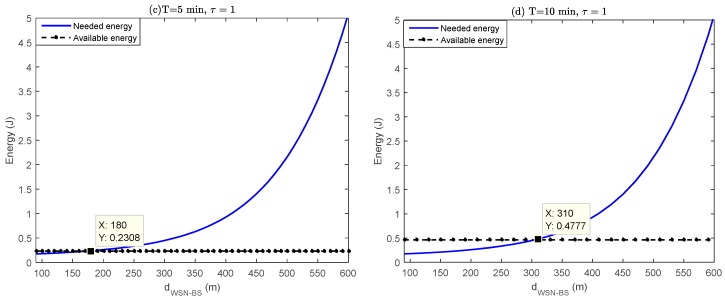
WSN performance.

Figure 20 shows the minimum distance at which the WSN should be deployed to the BS to enslave the sensor node to the recovered energy; it is observed that the range of the WSN is better when the controlled physical phenomenon varies slowly. More precisely:

- Figure 20a represents a WSN in which each round is performed every second; it appears that the node cannot operate because the recovered energy is insufficient.
- Figure 20b is the result of the case of the measures to be taken every minute; the energy harvested remains lower than the energy demand of the node.
- Figure 20c shows that the wireless should be deployed at only 180 m from the BS if each round lasts 5 min.
- If each round is performed every 10 min, then the BS should be located at 310 m from the wireless nodes (Figure 20c).

## 5. Conclusions

In this paper, a method for defining the specifications of a WSN powered by an REHS has been proposed. The proposed method is based on the performance enslavement of the WSN to the amount of ambient RF energy available. First, a comprehensive energy budget of the WNS, taking into account most of the dissipation sources, is established. The amount of energy required to operate the node is then compared to the amount of recoverable energy to define the WSN limits. To achieve optimum performance, methods to minimize the consumption of the sensor node are combined with techniques for optimizing REHS. 

More specifically, a highly sensitive, miniature and efficient rectifier circuit design methodology has been proposed. Given the low density of the surrounding power, the method consists of selecting the most sensitive diode. Diode HSMS 2850 of Avago and the VD topology were selected to achieve the best performance at the frequency of 2.45 GHz. The performance of the rectifier was then improved by designing a matching circuit. The gradient method search was used to optimize the components values. The simulated results were then validated experimentally, and were proven to lead to a very acceptable agreement. The designed circuit demonstrates a 2.3 V output DC voltage and a conversion efficiency of 71% for an RF input power of −2 dBm.

The electric output characteristics of the designed circuit were then used to enslave a sensor node, allowing it to overcome its dependence to the battery capacity. For this, the energy consumption of a sensor node in a WSN (LEACH protocol WSN) that equitably distributes its charges to all nodes was quantified. The proposed model takes into account several sources of dissipation that have not been considered in the literature. At 0 dBm of input power, it was shown that the WSN could be deployed up to 310 m from the BS when the controlled physical phenomenon changes every 10 min. These results are satisfactory for many applications of WSN in which the controlled physical phenomena vary slowly; this is, for example, the case of temperature variations due to thermal inertia.

While the results obtained in this work validate the use of electromagnetic waves as an alternative energy source for sensor nodes deployed in remote areas, it is still possible to surpass the results that were achieved. It would be valuable to consider radio modules operating in the GSM band rather than the ISM band since the 900 MHz GSM band has been known to have better power density. A propagation model of the signal in the coverage area would be valuable in developing new communication protocols that take into account the location of the node in the network; this would also allow an optimal task scheduling according to the amount of the available energy. To reduce the size of the rectenna, it would be interesting to use the integrated circuits, rather than the discrete components.

## Figures and Tables

**Table 1:** Recently used energy model.

Energy Sources	[20,21,22]	[23]	In This Work
Data acquisition	−	√	√
Communication	√	√	√
Data processing	√	√	√
Transient	−	√	√
Cluster formation	−	−	√

**Table 2:** Parameter values used in energy model.

Parameters	Symbol	Values
Network size	M×M	--
Number of node	N	--
Packet size	b	2000 [20]
Control packet size	$b_{1}$	200 [33]
Ratio of reception and Idle listening energy	β	0.85
Throughput of non-persistent CSMA	α	0.132 [Equation (7)]
Transmit amplifier free space	$\varepsilon_{fs}$	$7\mathrm{nJ}/\mathrm{bit}/m^{2}$ [23]
Transmit amplifier for two ray	$\varepsilon_{amp}$	$0.0013\;\mathrm{pJ}/\mathrm{bit}/m^{4}$ [28]
Energy dissipation: electronics	$E_{\mathrm{elec}}$	50 nJ/bit [28]
Distance CH to sink	$d_{2}$	≥88 m [Equation (3)]
Supply voltage to sensor	$V_{\sup}$	1.8 V [37]
Required current for sensing activity	$I_{\mathrm{sens}}$	25 mA [23]
Current: flash reading 1 byte data	$I_{\mathrm{read}}$	6.2 mA [31]
Current: flash writing 1 byte data	$I_{\mathrm{write}}$	18.4 mA [31]
Time duration for sensor node sensing	$T_{\mathrm{sens}}$	0.5 mS [23]
Time duration: flash reading	$T_{\mathrm{read}}$	565 μS [31]
Time duration: flash writing	$T_{\mathrm{writing}}$	12.9 mS [31]
Current: wakeup mode	$I_{A_{\mathrm{CM}}}$	8 mA [36]
Current: sleeping mode	$I_{S_{\mathrm{CM}}}$	1 μA [36]
Time duration of a round	T	--
Transmit data to BS	$T_{\mathrm{CH}-\mathrm{sink}}$	0.1 s [39]
Active time of the node	$T_{A_{\mathrm{CM}}}$	1 ms [34]
Sleeping time of the node	$T_{S_{\mathrm{CM}}}$	$T-T_{A_{\mathrm{CM}}}$
Active time of the CH	$T_{A_{\mathrm{CH}}}$	Equation (21)
Sleeping time of the CH	$T_{S_{\mathrm{CH}}}$	$T-T_{A_{\mathrm{CH}}}$
Time duration: sleep → Idle	$T_{\mathrm{transON}}$	2450 μs [34]
Time duration: Idle → sleep	$T_{\mathrm{transOFF}}$	250 μs [34]
Number of clock cycles per task	$N_{\mathrm{cyc}}$	$0.97\times 10^{6}$ [28]
Leakage current	$I_{0}$	1.196 mA [28]
Sensor frequency	f	191.42 MHz [13]
Constant depending on the processor	$n_{p}$	21.26 [28]
Thermal voltage	$V_{t}$	0.2 V [13]
Average Capacitance switched per cycle	$C_{\mathrm{avg}}$	7 pF [37]

**Table 3:** Commonly used Schottky Avago diodes.

Diodes	HSMS 2810 [42]	HSMS 2820 [43]	HSMS 2850 [44]	HSMS 2860 [45]
$C_{j0}\left(\mathrm{pF}\right)$	1.1	0.7	0.18	0.18
$R_{S}\left(\Omega \right)$	10	6	25	14
$V_{j}\left(V\right)$	0.65	0.65	0.35	0.65

$C_{j0}$ is the diode’s zero bias junction capacitance.

**Table 4:** Comparison of the proposed circuit with related design (experimental results).

Ref	Matching Filter	Input RF Power (dBm)	Maximum Conversion Efficiency (%)
[47]	Yes	10	66.8
[48]	Yes	8	25
This work	No	8	32
		10	34

**Table 5:** Components values for Figure 18.

Component Name	Value (Local Minimum Found)	Component Name	Value (Local Minimum Found)
$L_{1}$	4.80101 nH	$L_{5}$	2.50625 nH
$C_{1}$	15.2171 pF	$C_{5}$	26.7175 pF
$L_{2}$	14.8041 nH	$L_{6}$	48.9407 nH
$C_{2}$	6.8495 pF	$C_{6}$	3.5248 pF
$L_{3}$	6.2 nH	$L_{7}$	31.7322 nH
$C_{3}$	19.2243 pF	$C_{7}$	2.7 pF
$L_{4}$	48.2272 nH	$C_{8}$	2.7 pF
$C_{4}$	4.8669 pF	$R_{L}$	470 Ω

**Table 6:** Comparison of the proposed rectenna and related designs.

Ref (year)	Input RF Power for Maximum Conversion Efficiency (dBm)	Maximum Conversion Efficiency (%)	Type of Schottky Diode	Type of Result
[51] (2014)	0	42	SMS7630	Measured
[52] (2015)	0	65	MSS20-141	Simulated
[53] (2017)	12.9	56	HSMS 286 C	Simulated
[54] (2017)	5	65	HSMS 2850	Simulated
This Work (2017)	−2	71	HSMS 2850	Simulated
